# Cognitive Processing Efficiency (Throughput) Improves with Aerobic Exercise and Is Independent of the Environmental Oxygenation Level: A Randomized Crossover Trial

**DOI:** 10.3390/sports14010030

**Published:** 2026-01-07

**Authors:** Takehira Nakao, Toru Hirata, Takahiro Adachi, Jun Fukuda, Tadanori Fukada, Kaori Iino-Ohori, Miki Igarashi, Keisuke Yoshikawa, Kensuke Iwasa, Atsushi Saito

**Affiliations:** 1 Faculty of Human Science, Kyushu Sangyo University, 3-1 Matsukadai 2, Higashi-ku, Fukuoka 813-8503, Japan; 2 Faculty of Medicine, Miyazaki University, 5200 Kihara, Kiyotake-cho, Miyazaki 889-1692, Japan; toru_hirata@med.miyazaki-u.ac.jp; 3 Department of Pharmacology, Faculty of Medicine, Saitama Medical University, 38 Moro-hongo, Moroyama-machi, Iruma-gun, Saitama 350-0495, Japan; 4 Faculty of Human-Environment Studies, Kyushu University, 744 Motooka, Nishi-ku, Fukuoka 819-0395, Japan; saito-a@ihs.kyushu-u.ac.jp

**Keywords:** aerobic exercise, cognitive function, brain-vascular pathways, eicosapentaenoic acid, oxygen level

## Abstract

Aerobic exercise with eicosapentaenoic acid (EPA) may enhance cognition via cerebrovascular pathways. We tested whether mild hyperbaric oxygen (HBO; 1.41 atmospheres absolute [ATA], approximately 30% O_2_) adds to gains in cognitive processing capacity (throughput) versus normobaric normoxia (1.0 ATA, approximately 21% [20.9%] O_2_). Healthy young adults (*n* = 16) performed cycling exercise at 60–70% VO_2peak_ for 60 min, twice weekly, for 4 weeks per environment with a 1-week washout; EPA (2170 mg·day^−1^) was taken during each 4-week training phase (total 8 weeks) and was paused during the washout. An EPA-only control (*n* = 8) was included for supplementary analysis. The primary outcome was throughput (correct·min^−1^; T1–T4); secondary outcomes were interference indices (I1: stroop interference, I2: reverse-stroop interference). Effects were estimated using linear mixed models [environment, time, environment × time; AR(1), REML] and Hedges’ *g*_av_; accuracy used generalized estimating equations. Throughput improved mainly with time (T1–T2 *p* < 0.001; T4 *p* = 0.017; T3 *p* = 0.055), with no environment or interaction effects. I1/I2 showed no significant change, and one task exhibited an accuracy ceiling. Under safe, feasible conditions (≤1.41 ATA), aerobic exercise improved processing capacity (throughput) independently of environmental oxygenation level. The absence of detectable additive effects should be interpreted cautiously under conservative settings.

## 1. Introduction

Aerobic exercise enhances higher-order cognitive functions, such as attention and inhibitory control, by optimizing neurovascular units, primarily in the prefrontal cortex [1]. The hyperbaric oxygen (HBO) environment, which artificially modifies the available oxygen during exercise, increases the partial pressure of arterial blood oxygen and tissue oxygenation level, thereby influencing peripheral and cerebral circulation dynamics [2]. However, few studies have examined how exercise under hyperbaric oxygen (HBO) conditions—which can alter systemic and cerebral circulation—affects cognitive processing, including Stroop task throughput and interference. Furthermore, although eicosapentaenoic acid (EPA), an n-3 polyunsaturated fatty acid with anti-inflammatory and neuroprotective properties, may enhance these outcomes, its potential benefit when combined with HBO exercise remains largely unexplored. A recent preprint from our group (not yet peer-reviewed) reported that during exercise in an HBO environment, red blood cell count and hematocrit decreased whereas hemoglobin concentration remained stable, supporting a preliminary hypothesis that reduced blood viscosity and preserved oxygen-carrying capacity can coexist and may promote more efficient micro-circulation [3]. Furthermore, EPA supports neurovascular coupling reactivity by alleviating vascular inflammation and improving rheological properties [4,5]. Given these findings, HBO with exercise with EPA is expected to enhance the “functional reserve” of networks involved in conflict resolution and response selection, thereby boosting both the speed and accuracy of interference suppression. However, performance on the Stroop task is susceptible to the “speed-accuracy tradeoff,” and relying solely on reaction time or accuracy rates risks underestimating or overestimating the intervention effect [6]. Therefore, in the present trial we pre-specified throughput (correct responses per minute; correct·min^−1^) as the primary cognitive outcome, because it combines speed and accuracy into a single index of processing efficiency and is expected to be less susceptible to changes in the speed–accuracy tradeoff. In addition, The interference indices (I1/I2) and accuracy were analyzed as secondary outcomes to provide complementary information about where changes may emerge within the processing pipeline (perception, conflict resolution, and response selection) [7,8].

Furthermore, this study envisions future applications in space medicine. Astronauts perform tasks in spacesuits (typically 0.3 atm, 100% oxygen), effectively executing cognitive tasks in an environment equivalent to approximately 30% of terrestrial oxygen levels (ppO_2_ = PB × FiO_2_; where ppO_2_ is inspired oxygen partial pressure, PB is barometric pressure, and FiO_2_ is the fraction of inspired oxygen) [9]. However, the mild HBO condition used in this study is not intended to directly simulate such operational environments; thus, any potential applications to space medicine should be considered hypothetical, not directly supported by the present data, and interpreted with caution. This study provides foundational data to verify the effects of oxygen availability on cognitive function.

This study aimed to simultaneously examine aerobic exercise in an HBO environment and EPA intake within a randomized crossover framework in humans, clarifying their effects on speed-accuracy integrated performance (throughput) and speed-based interference (I1/I2). The primary hypothesis predicted that throughput would improve over time, regardless of environmental condition, reflecting the combined effects of exercise training and task learning. An exploratory hypothesis proposed that increased oxygen availability under HBO conditions might confer additional benefits, potentially appearing as an environmental main effect or an environment × time interaction. For the interference indices (I1/I2), we further hypothesized a directional decrease, with lower values indicating reduced interference, while expecting only small effect sizes given the short intervention period.

The present study provides three additions to the existing evidence base on mild HBO, exercise, and EPA supplementation, summarized in three points. First, in healthy young adults, we used a randomized crossover design to evaluate, within a single protocol, the combined effects of mild HBO exposure, aerobic exercise training, and EPA supplementation, thereby complementing prior studies that have often examined HBO or EPA separately and/or in clinical or mixed populations. Second, we selected throughput—a speed–accuracy integrated metric—as the primary cognitive outcome and used interference indices (I1/I2) as secondary measures, providing a processing-efficiency perspective beyond studies that often relied mainly on accuracy or reaction time alone. Third, we interpreted the observed cognitive changes within a physiological working model in which possible reductions in blood viscosity and preserved oxygen transport may influence cerebrovascular function, offering a hypothesis-generating framework that links exercise cognitive science with environmental physiology and nutritional science.

## 2. Materials and Methods

### 2.1. Study Design

**Figure 1:**
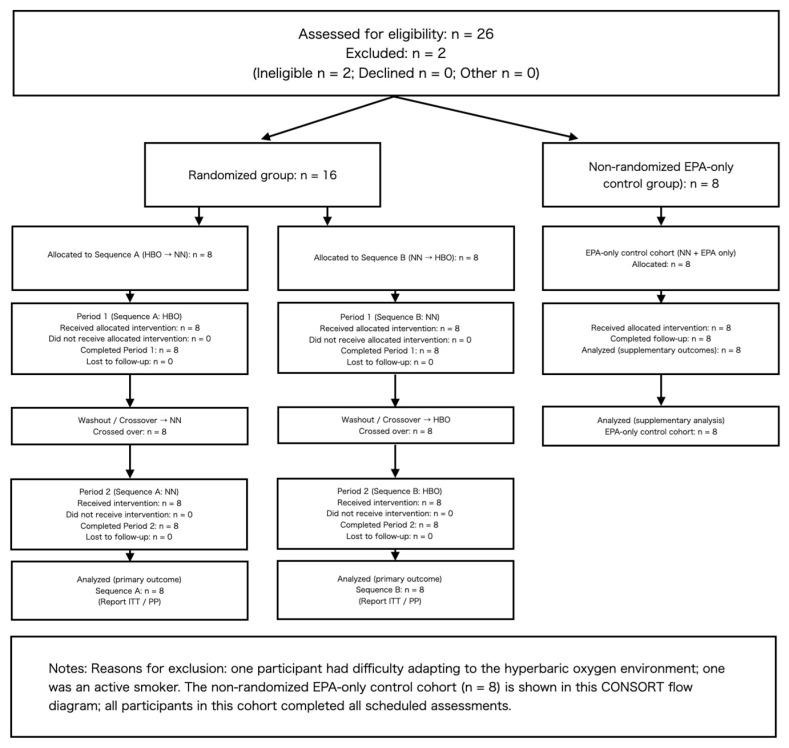
CONSORT flow diagram for the randomized two-period crossover trial with an additional non-randomized EPA-only control cohort. Twenty-six individuals were assessed for eligibility; two were excluded (one could not adapt to the hyperbaric oxygen environment; one was an active smoker). Sixteen participants were randomized (Sequence A: HBO → NN, *n* = 8; Sequence B: NN → HBO, *n* = 8), completed both periods, and were included in the primary analysis. A non-randomized EPA-only control cohort (NN + EPA only, *n* = 8) also completed all scheduled assessments and was included in secondary supplementary analyses.

**Figure 2:**
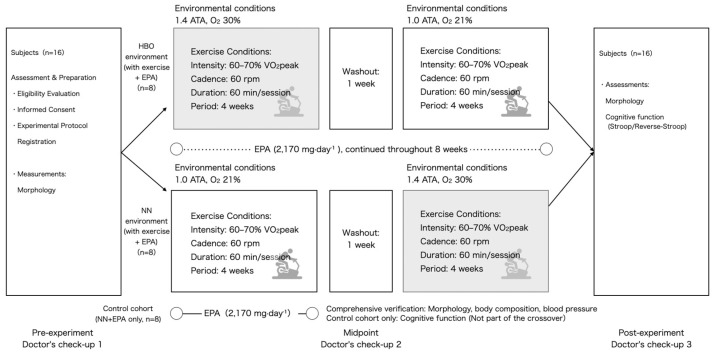
Randomized two-period crossover design. Participants (*n* = 16) completed two 4-week exercise phases under hyperbaric oxygen (HBO; 1.41 ATA, O_2_ 30%) or normobaric normoxia (NN; 1.0 ATA, O_2_ 20.9%) environments in a randomized order, separated by a 1-week washout. EPA supplementation (2170 mg·day^−1^) was taken during both 4-week phases (total 8 weeks) and paused during the washout. Assessments were performed before and after each phase (morphology, body composition, blood pressure, and cognitive function [Stroop/Reverse-Stroop]). The midpoint corresponds to the end of Phase 1 (Period 1 post) and, simultaneously, the start of Phase 2 (Period 2 pre) in the primary crossover analysis. Optional: A separate control cohort (NN + EPA only; non-randomized EPA-only group, *n* = 8) followed the same assessment schedule without crossover; this cohort was not part of the randomized crossover and is summarized in Supplementary Tables S1–S3. Oxygen fraction values in the schematic are rounded to the nearest integer; for example, 20.9% is shown as 21%. ATA, atmosphere absolutes; EPA, eicosapentaenoic acid.

This was a randomized crossover trial (Figure 1 and Figure 2) with a mixed design, combining a 2 × 2 within-subject crossover of HBO and normobaric normoxia (NN) with a parallel control group that received only EPA. Participants were divided into three groups. Two groups performed supervised cycling training under either HBO or NN conditions while simultaneously taking EPA daily: the HBO + exercise + EPA and the NN + exercise + EPA (randomized cross over 2 group). Subsequently, the crossover groups switched environments and repeated an identical protocol. The third group consumed only EPA without exercise and served as the EPA control group under the NN conditions (non-randomized EPA-only group). Each trial lasted for 4 weeks, with a 1-week washout period based on prior research on plasma EPA half-life [10,11,12]. Health examinations and measurements were conducted before, during, and after the experiments. This study was conducted in accordance with the principles of the Declaration of Helsinki and was approved by the Ethics Committee. Written informed consent was obtained from all the participants. The primary analysis focused on the crossover group (environment × time), whereas the EPA control group was included in the secondary analysis. This study was conducted as an experimental study on exercise and environmental physiology and not for medical purposes.

### 2.2. Participants

The participants were recruited between 25 April and 18 June 2024, through university bulletin boards and online announcements, including posts on social media. Twenty-four healthy adult males (mean ± standard deviation: age, 20.9 ± 1.4 years; height, 171.4 ± 5.6 cm; weight, 64.1 ± 11.6 kg; and body mass index (BMI), 21.8 ± 3.6 kg/m^2^) were randomly assigned to the HBO + exercise + EPA, NN + exercise + EPA, or EPA control groups (*n* = 8 per group). The inclusion criteria were as follows: no history of cardiovascular or metabolic disease, no history of smoking, ability to perform moderate-intensity exercise and take EPA-containing supplements during the study period, and ability to adapt to hyperbaric environments. The exclusion criteria were as follows: age < 20 years, excessive exercise habits, and requiring specific foods and/or supplements. Participants were instructed to avoid significant changes in their diet or lifestyle during the study period. The sample size estimation assumed a conventional medium effect size (f = 0.25) for a 2-group × 2-time point comparison in the absence of pilot data, using conservative assumptions (correlation among repeated measures = 0.50; ε = 1). It should be noted that this was a conservative setting for the crossover design that included within-subject comparisons; therefore, the sample size estimation should be interpreted cautiously.

### 2.3. Experimental Environment

#### 2.3.1. Environment

In the HBO environment, absolute pressure of 1.41 ± 0.01 ATA, oxygen concentration of 29–30%, temperature of 21.7 ± 0.7 °C, and humidity of 73.5 ± 4.6% were maintained. These settings were selected primarily based on the chamber’s certified operating limit for mild HBO operation (Type II; ≤1.41 ATA) and to remain within commonly used mild-HBO ranges reported in prior work [13]. The NN environment maintained an absolute pressure of 1.00 ± 0.01 ATA, an oxygen concentration of 20.9% (≈21%), a temperature of 22.1 ± 1.1 °C, and a humidity of 70.5 ± 6.5%. The HBO environment was constructed in an artificial environmental control chamber (Japan Pressure Bulk Industries Co., Ltd., Shizuoka, Japan) at a pressurization/depressurization rate of 0.07 ATA/min. A bicycle ergometer (STB-3400; Nihon Kohden Co., Ltd., Tokyo, Japan) was installed inside the chamber. The NN environment is established in a standard laboratory equipped with a similar facility. The hyperbaric chamber used complied with domestic operational safety standards (mild HBO Type II, ≤1.41 ATA) and was distinguished from medical treatment devices.

#### 2.3.2. Eligibility Assessment, Pre-Experimental Measurements, and Medical Examination

Before enrolling for the experiment, the participants underwent a preliminary interview, supervised cycling training (peak oxygen uptake [VO_2peak_] measurement), and an assessment of their ability to adapt to the HBO environment. Medical examinations were performed by a physician. All outcome measures were assessed at three time points: baseline (before the first 4-week intervention phase), immediately after the first 4-week phase (before the 1-week washout), and after completion of the second 4-week phase. In the primary crossover analysis, the midpoint assessment served as the Period 1 post value and the Period 2 pre value and was not treated as an independent endpoint.

#### 2.3.3. Anthropometric Measurements

Height was measured using a standard height gauge (A&D Co., Ltd., Tokyo, Japan). Weight and body composition parameters (fat-free mass [FFM], fat mass [FM], and fat mass percentage [% FM]) were assessed using a dedicated bioelectrical impedance analyzer (InBody 770; InBody Japan Co., Ltd., Tokyo, Japan). Based on these measurements, the BMI was calculated using the following formulas: BMI = weight (kg)/height^2^ (m^2^). Systolic blood pressure and diastolic blood pressure were measured using an automatic upper arm blood pressure monitor (HBP-1300; Omron Co., Ltd., Kyoto, Japan) via an oscillometric method.

#### 2.3.4. Exercise Load

The exercise intensity for the bicycle ergometer test was set at 60–70% of the VO_2peak_. The pedaling speed was 60 rpm, and each session lasted 60 min. The test was conducted twice a week for 4 weeks. Before the intervention, VO_2peak_ was estimated using the breath-by-breath method with a respiratory metabolic monitoring system (AE-310S; Minato Medical Science Co., Ltd., Osaka, Japan). Exercise in the HBO environment was performed for 60 min after reaching the predetermined absolute pressure. The time required for pressurization and decompression was approximately 20 min. Participants in the HBO group exercised in the HBO environment during the first phase and in the ambient-pressure environment during the second phase, while those in the ambient-pressure group exercised in the reverse order. All exercise sessions were conducted in person under direct supervision by research staff, and adherence was monitored through individual training logs documenting attendance at each scheduled session. All participants completed 100% of their prescribed sessions. The effects of the sequence and timing were examined using sensitivity analyses in the statistical analysis.

#### 2.3.5. EPA Intake

During the study period, participants were instructed to take a high-concentration EPA supplement (Bizen Chemical Co., Ltd., Akaiwa, Japan) at 2170 mg daily in capsule form. The EPA intake overlapped with the exercise period in both environments and was suspended before study initiation and during the 1-week washout. Adherence was monitored by capsule counts from pre-packed supplies and by a daily online log in which participants recorded intake status, gastrointestinal symptoms, and stool characteristics. The EPA dosage was food-grade and within the recommended range for dietary reference intake in Japan [14].

#### 2.3.6. Cognitive Function Assessment: Throughput, T1–T4, and I1–I2

The New Stroop Test II (Toyophysical Co., Ltd., Fukuoka, Japan) was used to administer four tasks: T1 Reverse Stroop Control (semantic selection with no interference), T2 Reverse Stroop Interference (semantic selection with color ink interference), T3 Stroop Control (color name selection with no interference), and T4 Stroop Interference (color name selection with semantic interference) [15,16]. Each block was a 60 s self-paced response task with a randomized stimulus presentation and counterbalanced block order. Primary outcomes were throughput (correct responses per minute; correct·min^−1^), integrating speed and accuracy; secondary outcomes included interference indices (I1/I2); and reference outcomes include accuracy (correct response rate). Throughput (T1–T4) was calculated as the number of correct responses in each 60 s block (errors and omissions excluded) and expressed as correct responses per minute (correct·min^−1^). Interference indices were defined as I1 = (T3 − T4)/T3 and I2 = (T1 − T2)/T1. Accuracy was defined as a correct/incorrect response. Interpretation was based on both the *p*-value (Holm-corrected) and effect size [Hedges’ *g*_av_·95% CI]. Positive values indicate improved throughput (increase), whereas decreases in I1/I2 indicate reduced interference. All cognitive measurements were conducted under NN immediately before and after each 4-week training phase. The testing environment was standardized to NN to isolate on the long-term effects of regular training rather than acute HBO exposure, and because administering the full cognitive protocol inside the HBO chamber was not feasible for practical and safety reasons.

#### 2.3.7. Statistical Analysis

Analyses were performed using IBM SPSS Statistics ver.27 (IBM Corp., Armonk, NY, USA). The primary (throughput) and secondary outcomes (I1/I2) were analyzed using linear mixed models (LMM). Fixed effects included environment (HBO/NN), time (Post/Pre), and their interaction. Random effects included the participant ID intercept. The repeated-measures covariance structure was first-order autoregressive (AR(1)), and the estimation was based on the restricted maximum likelihood (REML). Assumptions of the LMMs (normality and homoscedasticity of residuals) were evaluated using Q–Q plots and residual-versus-fitted plots, and no meaningful deviations were observed. Fixed effect tests used Type-III Wald χ^2^ (df = 1), and estimates were reported as coefficient ± standard error (SE) and 95% confidence interval (CI). The effect size for within-group change was determined using Hedges’ *g*_av_ (95% CI). The reference outcome (accuracy) was analyzed using generalized estimating equations (GEE; binomial logit, exchangeable, and robust SE). Effect measures are reported as odds ratios (ORs) and 95% CI. Multiplicity was corrected using Holm’s method. The significance level was set at α = 0.05 (two-tailed), and no additional exploratory significance threshold (e.g., *p* < 0.10) was prespecified; *p*-values above 0.05 were interpreted descriptively. Because the sample size (*n* = 16) was constrained by practical and budgetary factors, a post hoc G*Power 3.1 calculation for a 2 (environment: HBO vs. NN) × 2 (time: pre vs. post) repeated-measures analysis of variance (within–between interaction), assuming a correlation of 0.50 among repeated measures (ε = 1), indicated adequate power (1–β > 0.80) only for large effects (approximately f = 0.50). Detecting a medium effect (f = 0.25) would require 34 participants in total. This study should therefore be considered exploratory, with limited power for small-to-moderate effects, and results were interpreted with greater emphasis on estimated coefficients, their 95% confidence intervals (CIs), and effect sizes rather than *p*-values alone.

## 3. Results

### 3.1. Summary

**Figure 3:**
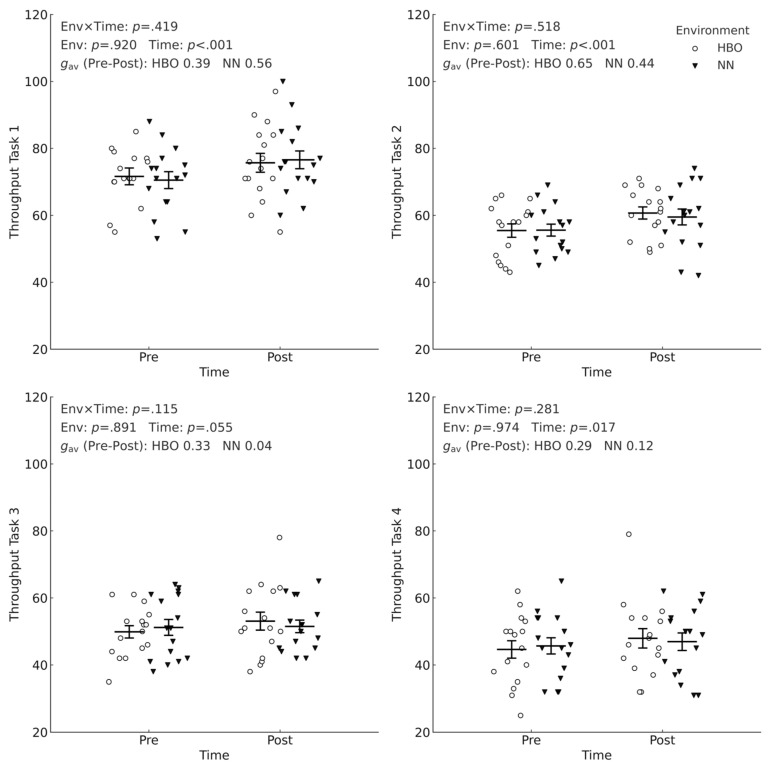
Throughput performance in Stroop tasks (T1–T4) by environment (HBO vs. NN) and time (pre vs. post). Short horizontal bars indicate group means and T-shaped error bars indicate the standard error. Symbols represent individual participant values with minor horizontal jitter for visibility. White circles, HBO; black triangles, NN. Throughput, correct·min^−1^. HBO, hyperbaric oxygen; NN, normobaric normoxia.

The environment × time interaction and the main effect of the environment were not significant. However, the main effect of time was significant at T1 (*p* < 0.001, 95% CI 2.63–7.49), T2 (*p* < 0.001, 95% CI 2.59–6.59), T4 (*p* = 0.017, 95% CI 0.40–4.16), whereas the time effect at T3 did not reach the predefined significance level (*p* = 0.055, 95% CI −0.03–3.53; Figure 3). Across all tasks (T1–T4), accuracy was already very high and showed minimal variation across environments and time points. Accuracy in Task 3, in particular, was 100% in all conditions (Supplementary Table S2).

### 3.2. Physical Characteristics

Table 1 shows the descriptive statistics (mean ± standard deviation) for each group before and after the intervention. The interaction effect of the environmental conditions and time was not significant for any of the following variables: height, weight, BMI, body composition parameters, or blood pressure. No significant changes in the physical characteristics were observed in the EPA control group (all *p* > 0.050).

### 3.3. Cognitive Processing Efficiency (Throughput)

Figure 3 shows the pre- and post-intervention throughputs of the HBO + exercise + EPA group and the NN + exercise + EPA group. No interactions were observed across any of the four tasks (T1: *p* = 0.419, T2: *p* = 0.518, T3: *p* = 0.115, T4: *p* = 0.281), and the main effect of the environment was also nonsignificant (T1, *p* = 0.920, T2, *p* = 0.601, T3, *p* = 0.891, T4, *p* = 0.974). In contrast, the main effect of time was significant at T1 and T2 (both *p* < 0.001), significant at T4 (*p* = 0.017), and did not reach significance at T3 (*p* = 0.055), indicating that the throughput improved after the intervention regardless of the environment. The magnitude of the pre- and post-intervention difference (Hedges’ *g*_av_) varied by task: T1-HBO, 0.39; NN, 0.56; T2-HBO, 0.65; NN, 0.44; T3-HBO, 0.33; NN, 0.04; T4-HBO, 0.29; and NN, 0.12. According to conventional benchmarks (0.2, 0.5, and 0.8 representing small, medium, and large effects, respectively), the improvements in throughput at T1 and T2 can be interpreted as small to medium in magnitude—particularly in NN at T1 (*g*_av_ = 0.56) and HBO at T2 (*g*_av_ = 0.65)—whereas the effects at T3 and T4 were small or trivial regardless of environment. Figure 3 shows individual data points and estimated marginal means ± SE, with units expressed as correct·min^−1^.

#### 3.3.1. T1–T4 and I1–I2: LMM Test Table

In the LMM for T1–T4 based on unstandardized throughput values (correct·min^−1^), no environment×time interaction was detected (Table 2) (T1: χ^2^ = 0.654, *p* = 0.419; T2: χ^2^ = 0.417, *p* = 0.518; T3: χ^2^ = 2.491, *p* = 0.115; T4: χ^2^ = 1.161, *p* = 0.281). No main effect of the environment was observed either (T1: χ^2^ = 0.010, *p* = 0.920; T2: χ^2^ = 0.274, *p* = 0.601; T3: χ^2^ = 0.019, *p* = 0.891; T4: χ^2^ = 0.001, *p* = 0.974). On the other hand, the main effect of Time (post vs. pre) on throughput was significant at T1 (coefficient = 5.062 correct·min^−1^, 95% CI 2.63–7.49, χ^2^ = 16.757, *p* < 0.001), T2 (coefficient = 4.594 correct·min^−1^, 95% CI 2.59–6.59, χ^2^ = 20.457, *p* < 0.001), and T4 (coefficient = 2.281 correct·min^−1^, 95% CI 0.40–4.16, χ^2^ = 5.682, *p* = 0.017), whereas the time effect at T3 did not reach the predefined significance level (coefficient = 1.750 correct·min^−1^, 95% CI −0.03–3.53, χ^2^ = 3.692, *p* = 0.055).

#### 3.3.2. T1–T4 and I1–I2: Effect Sizes Within the Same Environment

For within-environment changes (Hedges’ *g*_av_), throughput improvements were largest at T2 (Table 3), with a clear improvement also at T1, whereas T3 and T4 showed at most small and imprecise effects. Using conventional benchmarks (0.2, 0.5, and 0.8 representing small, medium, and large effects, respectively), the effect sizes for HBO were small at T1 (*g*_av_ = 0.39, 95% CI 0.05–0.74), medium at T2 (0.65, 0.42–0.88), and small at T3 (0.33, −0.01–0.67) and T4 (0.29, −0.01–0.58). For NN, the effects were medium at T1 (0.56, 0.34–0.78), small to medium at T2 (0.44, 0.12–0.77), and trivial to small at T3 (0.04, −0.22–0.28) and T4 (0.12, −0.10–0.33). Overall, T1 and T2 showed small-to-medium improvements in throughput—particularly the moderate-to-large effects at T2 in HBO and at T1 in NN—whereas T3 and T4 showed at most small effects. For T3 and T4 in both environments, however, the 95% CIs frequently included zero, indicating substantial uncertainty and suggesting that these small effects should be interpreted with caution.

The effect sizes for the interference indices (I1/I2) were close to zero with wide CIs, indicating no meaningful changes in interference control in either environment (HBO: I1 = −0.09, −0.57–0.39, I2 = 0.09, −0.29–0.47; NN: I1 = 0.14, −0.50–0.78, I2 = −0.08, −0.52–0.37).

## 4. Discussion

This study used a randomized crossover design to examine the effects of supervised cycling training under an HBO/NN environment with concurrent EPA supplementation on processing efficiency (throughput) and interference indices (I1/I2) in Stroop-type cognitive tasks. First, although throughput consistently improved with time, the main effect of the environment and the environment × time interaction were not significant, and no short-term additive effect of EPA was observed. Furthermore, I1/I2 showed no significant changes, and the accuracy in one task was 100% across all conditions (Supplementary Table S2), suggesting a potential ceiling effect given the already very high accuracy and minimal variation across environments and time points. In practical terms, a higher throughput means that participants produced more correct responses in the same amount of time without losing accuracy, indicating an improvement in the overall processing efficiency in this Stroop-type task. Therefore, the observed enhancement in processing efficiency can be interpreted as primarily resulting from the progressive integration and optimization of speed and accuracy (semi-automation of response selection and efficient allocation of attentional resources) through regular aerobic exercise and task learning (repetition) [17,18,19], although potential influences such as placebo and expectation effects cannot be completely ruled out. At the same time, part of the time-related increase in throughput is likely attributable to task learning due to repeated exposure to the same Stroop task, as well as to inter-individual variability in responsiveness. This supports the notion that exercise produces generalizable facilitation across the processing pipeline and improves the efficiency of the entire Stroop task processing pipeline (perception to conflict resolution to response selection). Specifically, both behavioral measures (RT reduction and accuracy maintenance) and event-related potentials indicate that after aerobic exercise, resource allocation increases in the perception/stimulus evaluation stage (P2/P3), responsiveness to control demands in the conflict/inhibition stage (N2) improves, and consequently, response selection stabilizes and accelerates [20,21,22].

However, these findings do not imply that HBO is ineffective. Potential reasons for failing to detect additional benefits of HBO include that participants were healthy young adults with high baseline abilities and limited room for improvement, as reflected in the generally high accuracy and throughput levels observed in this study [23,24,25]. Second, the possibility that the mild HBO settings (≤1.41 ATA), exercise intensity and frequency, and EPA dosage and duration did not reach physiological effect thresholds should be viewed as a hypothesis generated from this protocol, requiring direct physiological verification. Third, given the specific structure of the Stroop-type task used here—which may have been dominated by visual search and response selection—differences in oxygen availability seem unlikely to have meaningfully influenced the interference indices. The primary implication of this study is that improvements in processing efficiency can be achieved through exercise training and task repetition, independent of the environment. Therefore, any additive effects of HBO and/or EPA may have been undetectable within the doses, durations, test conditions, sample size, and evaluation metrics used in this study. Furthermore, this trial has methodological significance owing to its establishment and validation of protocol and measurement system (HBO settings, pressurization rate, exercise modality, and throughput as primary outcomes) that can be safely implemented in humans.

Furthermore, the lack of a significant change in I1/I2 suggests that interference control did not improve meaningfully in either environment. Several methodological factors may explain this null finding. First, task learning may initially enhance processing speed and only later affect interference suppression. Second, the evaluation window (block length or measurement timing) may have been insufficient for detecting subtle changes, and the sensitivity of I1/I2 may have been limited under the present task conditions. Third, variance in the metric may have been constrained by ceiling or floor effects [26,27,28]. Fourth, interference indices may be relatively stable and may require a longer intervention duration or greater task difficulty to detect changes. This interpretation should be considered a methodologically grounded hypothesis that should be examined explicitly in future studies. Future work requires optimization of the interference intensity (incongruity ratio, stimulus probability, and conflict parameters) and task difficulty, along with refinement of the measurement design (block length and retest timing). In summary, the findings of this study can be coherently explained using a biphasic model. While motor adaptation (enhancement of efficiency in neurovascular units centered on the prefrontal cortex) and task learning (optimization of stimulus-response mapping) drive throughput increases, HBO/EPA likely has minimal effects or remains within the range of measurement sensitivity under the current conditions and participants. Based on prior literature, the proposed mechanisms may include three partly overlapping pathways. First, cerebral blood flow and neurovascular coupling may become more responsive to task demands [17,18,19]. Second, lactate signaling and mobilization of alternative fuels, such as medium-chain triglycerides, may stabilize substrate supply during short-duration, high-demand tasks [29,30,31]. Third, autonomic nervous system regulation, indexed by heart rate variability (HRV), may reduce reaction time variability and support more consistent performance [32,33,34]. However, these mechanisms are inferred from prior work rather than directly demonstrated here because physiological indicators were not measured concurrently. Therefore, they remain hypothetical and require direct verification through simultaneous physiological monitoring in subsequent research. Thus, our mechanistic interpretation should be regarded as hypothesis-generating. This study has several methodological limitations. First, the relatively small sample size reduced the statistical power of the data to detect small environmental and interaction effects. Second, complete blinding to the environmental conditions was not feasible because the participants could perceive changes in barometric pressure via the inner ear. Third, despite the randomized crossover design and washout period, order effects between the HBO and NN conditions could not be completely ruled out. Therefore, the present findings should be interpreted as preliminary and hypothesis-generating.

Immediate priorities for future research should include direct assessment of neurovascular–metabolic coupling through simultaneous measurements, such as functional near-infrared spectroscopy, electroencephalography, and HRV, as well as broadening the study population to include older adults, at-risk groups, and women. Additional work should optimize dose, duration, and frequency across all components of the intervention, including HBO parameters (partial pressure of oxygen, exposure time, and session frequency); exercise frequency, intensity, time, and type; EPA dose, and intervention duration. Further studies should also increase task difficulty to avoid ceiling effects (manipulating the mismatch ratio, stimulus presentation probability, and competition intensity) and refine the assessment timing to distinguish acute responses, delayed effects, and longitudinal changes. This research program of research should be systematically advanced to rigorously test the boundary conditions for additive effects—specifically, which doses, durations, and loads elicit them—and to evaluate the validity of the proposed mechanisms [1,35,36], thereby strengthening the external validity of the protocol established in this study.

## 5. Conclusions

This randomized crossover study examined the combined effects of supervised cycling training under HBO and NN environments with concurrent EPA supplementation on processing efficiency (throughput) and interference indices (I1/I2) in Stroop-type cognitive tasks in healthy young men. Throughput improved over time irrespective of the environmental condition, whereas no additional short-term cognitive benefit of mild HBO and/or EPA was detected under the present protocol and I1/I2 remained unchanged. These findings indicate that under the present conditions with concurrent EPA supplementation, regular aerobic exercise under NN can induce modest improvements in time-pressured information processing, whereas any additive cognitive effect of mild HBO was, at most, too small to be detected in this cohort and setting.

## Figures and Tables

**Table 1:** Participant characteristics before and after the intervention in the HBO + exercise + EPA group, the NN + exercise + EPA group, and the NN + EPA group.

		HBO + Ex + EPA			NN + Ex + EPA			NN + EPA		*p* Value Environment × Time Interaction
Measurement		Pre	Post		Pre	Post		Pre	Post	
Height	cm	170.4 ± 5.9	170.3 ± 5.9		170.7 ± 6.0	170.4 ± 5.9		172.9 ± 5.0	172.6 ± 5.2	0.113
Weight	kg	65.1 ± 11.4	65.8 ± 11.8		64.5 ± 12.0	64.9 ± 11.0		63.3 ± 12.3	63.5 ± 12.3	0.588
BMI	kg/m^2^	22.3 ± 2.9	22.5 ± 3.1		22.0 ± 3.3	22.3 ± 2.9		21.2 ± 4.5	21.4 ± 4.6	0.975
FFM	kg	52.2 ± 6.9	52.7 ± 7.2		52.3 ± 7.3	52.0 ± 6.5		55.0 ± 7.8	54.6 ± 7.1	0.108
FM	kg	12.9 ± 5.3	13.1 ± 5.5		12.2 ± 5.6	13.0 ± 5.5		8.3 ± 5.6	8.9 ± 5.8	0.260
%Fat	%	19.2 ± 4.8	19.3 ± 4.9		18.3 ± 4.8	19.4 ± 5.1		12.4 ± 5.1	13.2 ± 5.2	0.137
SBP	mmHg	115.7 ± 9.6	117.6 ± 7.6		116.4 ± 7.3	114.2 ± 9.5		115.0 ± 10.9	114.8 ± 11.2	0.107
DBP	mmHg	61.0 ± 5.9	61.4 ± 6.8		60.1 ± 7.0	61.3 ± 6.9		63.1 ± 9.7	61.9 ± 7.0	0.806

**Table 2:** The Type-III Wald χ^2^ (df = 1) test results for fixed effects in the LMM.

Task	Effect	Coef	SE	95% CI (Low)	95% CI (High)	Wald χ^2^(1)	*p*
T1	Env × Time	2.000	2.47	−2.84	6.84	0.654	0.419
	Environment	−0.125	1.24	−2.56	2.31	0.010	0.920
	Time	5.062	1.24	2.63	7.49	16.757	*p* < 0.001
T2	Env × Time	−1.312	2.03	−5.29	2.67	0.417	0.518
	Environment	−0.531	1.02	−2.53	1.47	0.274	0.601
	Time	4.594	1.02	2.59	6.59	20.457	*p* < 0.001
T3	Env × Time	−2.875	1.82	−6.44	0.69	2.491	0.115
	Environment	−0.125	0.91	−1.91	1.66	0.019	0.891
	Time	1.750	0.91	−0.03	3.53	3.692	0.055
T4	Env × Time	−2.062	1.91	−5.81	1.68	1.161	0.281
	Environment	0.031	0.96	−1.85	1.91	0.001	0.974
	Time	2.281	0.96	0.40	4.16	5.682	*p* = 0.017
I1	Env × Time	1.750	2.47	−3.08	6.58	0.503	0.478
	Environment	1.500	1.23	−0.92	3.92	1.479	0.224
	Time	0.188	1.23	−2.23	2.60	0.023	0.879
I2	Env × Time	−1.500	2.27	−5.95	2.95	0.436	0.509
	Environment	1.000	1.14	−1.23	3.23	0.776	0.378
	Time	0.063	1.14	−2.16	2.29	0.003	0.956

**Table 3:** The effect sizes (Hedges’ *g*_av_) before and after exposure to the same environment.

Env.	Task	*g*_av_ (Post-Pre)	95% CI (Low)	95% CI (High)
HBO	T1	0.39	0.05	0.74
	T2	0.65	0.42	0.88
	T3	0.33	−0.01	0.67
	T4	0.29	−0.01	0.58
	I1	−0.09	−0.57	0.39
	I2	0.09	−0.29	0.47
NN	T1	0.56	0.34	0.78
	T2	0.44	0.12	0.77
	T3	0.04	−0.22	0.28
	T4	0.12	−0.10	0.33
	I1	0.14	−0.50	0.78
	I2	−0.08	−0.52	0.37

## Data Availability

The data sets used and analyzed during the current study are available from the corresponding author on reasonable request.

## Supplementary Materials

The following supporting information can be downloaded at: https://www.mdpi.com/article/10.3390/sports14010030/s1, Table S1: Model–based estimated marginal means (EMM) with 95% CIs by Environment (HBO/NN), Time (Pre/Post), and Task (T1–T4, I1–I2); Table S2: Accuracy (A1–A4): GEE odds ratios (Post vs Pre) and Type–III Wald tests; Table S3: Control cohort (NN+EPA): pre–post descriptive statistics, Hedges’ gav (95% CI) for continuous outcomes (T1–T4, I1–I2), and GEE odds ratios for accuracy (A1–A4).

## Abbreviations

ATA	atmosphere absolute
BMI	body mass index
DBP	diastolic blood pressure
EPA	eicosapentaenoic acid
FFM	fat-free mass
FM	fat mass
HBO	hyperbaric oxygen
NN	normobaric normoxia
O_2_	oxygen
VO_2peak_	peak oxygen uptake
%FM	percent fat mass
I1	stroop interference
I2	reverse-stroop interference
SBP	systolic blood pressure
